# The Enterovirus Theory of Disease Etiology in Myalgic Encephalomyelitis/Chronic Fatigue Syndrome: A Critical Review

**DOI:** 10.3389/fmed.2021.688486

**Published:** 2021-06-18

**Authors:** Adam J. O'Neal, Maureen R. Hanson

**Affiliations:** Department of Molecular Biology and Genetics, Cornell University, Ithaca, NY, United States

**Keywords:** myalgic encephalomyelitis, chronic fatigue syndrome, enterovirus, chronic infection, RT-PCR, serology, immunohistochemistry, cell culture

## Abstract

Myalgic Encephalomyelitis/Chronic Fatigue Syndrome (ME/CFS) is a complex, multi-system disease whose etiological basis has not been established. Enteroviruses (EVs) as a cause of ME/CFS have sometimes been proposed, as they are known agents of acute respiratory and gastrointestinal infections that may persist in secondary infection sites, including the central nervous system, muscle, and heart. To date, the body of research that has investigated enterovirus infections in relation to ME/CFS supports an increased prevalence of chronic or persistent enteroviral infections in ME/CFS patient cohorts than in healthy individuals. Nevertheless, inconsistent results have fueled a decline in related studies over the past two decades. This review covers the aspects of ME/CFS pathophysiology that are consistent with a chronic enterovirus infection and critically reviews methodologies and approaches used in past EV-related ME/CFS studies. We describe the prior sample types that were interrogated, the methods used and the limitations to the approaches that were chosen. We conclude that there is considerable evidence that prior outbreaks of ME/CFS were caused by one or more enterovirus groups. Furthermore, we find that the methods used in prior studies were inadequate to rule out the presence of chronic enteroviral infections in individuals with ME/CFS. Given the possibility that such infections could be contributing to morbidity and preventing recovery, further studies of appropriate biological samples with the latest molecular methods are urgently needed.

## Introduction

Myalgic encephalomyelitis/chronic fatigue syndrome (ME/CFS) is a complex multi-system disease of unknown cause for which there is little insight into the molecular basis of disease progression, persistence and in rare cases - remission. The ME/CFS literature includes findings of patient immune system irregularities, abnormal cellular energy metabolism, and various altered autonomic nervous system manifestations including post-orthostatic tachycardia syndrome, orthostatic intolerance, and dysregulated hypothalamus pituitary adrenal axis. A hallmark symptom, required for many case definitions, is exercise intolerance or post-exertional malaise (PEM) (1, 2). The name of the illness itself is controversial, with one view holding that Myalgic Encephalomyelitis, a name dating from a series of early outbreaks of the disease (3), defines an illness that is different than Chronic Fatigue Syndrome, a name created in 1988 through a U.S. government committee (4). A discussion of the case definition and nomenclature is outside of the scope of this article, so we will use “ME/CFS” despite of the possibility that the initial CFS case definition results in inclusion of individuals who would not have met earlier criteria for Myalgic Encephalomyelitis.

ME/CFS case documentation shows evidence of both sporadic events involving singular individuals and regional outbreaks involving significant fractions of affected communities, especially hospitals, schools, and military bases. Machine learning estimation of ME/CFS prevalence using large-scale medical claims data gives a frequency of diagnosis in the United States that falls somewhere between 1.7 and 3.38 million Americans (5) and world-wide, the prevalence may be as high as 65 million (6). ME/CFS is not a rare disease and therefore understanding of disease pathophysiology and discovery of standardized biological markers or tests are important to identify appropriate treatments.

The pattern of transmissibility, and acute symptom constellation reminiscent of a flu-like illness, led early investigators to hypothesize a viral theory of ME/CFS disease etiology. Indeed, a number of researchers have interrogated a diverse range of microbial pathogens as triggers and/or perpetuators of the ME/CFS disease state. These include but are not limited to Epstein-Barr virus, cytomegalovirus, parvovirus B19, Brucella, Toxoplasma, *Coxiella burnetti, Chlamydia pneumoniae*, human herpesviruses, enteroviruses, human T cell leukemia virus II-like virus, spumavirus, hepatitis C virus, and human lentiviruses (7–9).

Between the 1930s and 1960s, a number of globally occurring ME/CFS outbreaks, with a spatiotemporal incidence coinciding with poliovirus epidemics, appeared under the titles of “abortive or atypical poliomyelitis” transitioning to “benign myalgic encephalomyelitis” or “epidemic neuromyasthenia” as physicians sought a term to describe the symptom profile of affected individuals (3, 10, 11). A ME/CFS outbreak occurred in 1934 California and provides a representative example of clinical features experienced by patients during similar epidemics of the time. Briefly, the 1934 outbreak occurred among roughly 200 hospital employees, primarily female, who fell ill with what acutely appeared to be poliomyelitis. Epidemiological deviations from what is commonly expected in poliomyelitis epidemics included relatively high attack rates, low mortality rates, low paralytic rates and a high incidence in adults as opposed to young children. Symptoms of sufferers included significant diurnal temperature fluctuations, localized muscular weakness as well as pain and muscle tenderness. Patients further exhibited numbness, paresthesia, exercise intolerance, and recurrent systemic and neurological symptoms. Longitudinal tracking of a subset of these patients showed residual muscle alterations, fatigue, and mental changes. Electromyograms showed generalized, mild, motor neuron changes and observations indicated that recurrences could occur even after many years of relatively normal health (10, 12). The totality of these findings indicated an infectious agent although tests available at the time could not convincingly implicate a specific culprit.

Subsequent outbreaks displayed the same basic features of the 1934 outbreak with some distinct clinical presentations depending on the region (3, 11, 13). Overall, most epidemic outbreaks have occurred in mid-spring through early fall indicating a virus with seasonal epidemic trends may be involved. Seasonality is not rare for viruses; many types, including but not limited to echovirus, coxsackievirus and poliovirus-related species, are well-known to have strong outbreak seasonality peaking in the month of August or early fall (10, 14). Outbreaks occurring after 1934 that deserve notable mention based on similar clinical presentations and links to an enteroviral culprit are highlighted below:

- 1949–1953 Adelaide, Australia: Dr. R. A. Pellew conducted several animal studies using patient throat washings, feces and cerebrospinal fluid collected from the 1949–1953 Adelaide outbreak as inoculants into rhesus monkeys, rabbits, mice, and hen eggs. Investigation into two monkeys repeatedly inoculated with patient sample revealed minute red spots along the course of the sciatic nerve, infiltration of lymphocytes and mononuclear cells into nerve roots and nerve fibers showing patchy damage to the myelin sheath with axon swelling. Although similar to poliovirus inoculation outcomes, these monkeys displayed more widespread changes in additional areas of the nervous system with no evidence of damage to nerve cells in the brain and spinal cord. Additionally, severe myocarditis was found in one of the two monkeys studied – myocarditis being most commonly caused by enteroviruses (10, 15).
- 1948 Akureyri, Iceland: Incidence of over 1,000 cases during a 3 month period resulted in the naming of “Icelandic disease,” which would later evolve to “benign myalgic encephalomyelitis” (16). Those who fell ill with the disease showed classical viral-type illness onset which later developed into a systemic form of the illness with symptoms including low fever and significant muscle tenderness/weakness. Due to the occurrence of concurrent local poliomyelitis epidemics, infectious disease testing was conducted but failed to indicate poliovirus, coxsackievirus or other known encephalitis viruses (10).
- 1956 Thorshofn/Egilsstadir, Iceland: Differential poliovirus vaccination responses between children exposed verses unexposed to the “Icelandic disease” indicated the etiological agent in ME/CFS may be a virus immunologically related to poliovirus. Children in a northeastern village of Iceland, Thorshofn, generated a slight rise in antibody production following vaccine administration whereas children from Egilsstadir, roughly 200 km south, had a much stronger immune response to polio vaccine administration. The difference between the two locations was that children from Eglisstadir were from an area which recently experienced a myalgic encephalomyelitis outbreak whereas children from Thorshofn were not (17). This indirect evidence of unknown prior immunity was also noted in the aforementioned Adelaide outbreak. This was evidenced by a 43% reduction in polio cases in the south of Australia, where Adelaide is located, compared to regions such as New South Wales and Queensland that reported increased polio cases (18). Enteroviral cross-immunity is well-documented in the enterovirus field and suggests that children in ME/CFS affected areas had been exposed to an agent immunologically similar to poliovirus (19).

Similar epidemic events of ME/CFS have occurred globally over time where patients display acute symptoms are similar to some poliomyelitis-afflicted patients. The later phases of disease progression make evident several differences between ME patients and those with poliomyelitis. The occurrence of considerable symptom constellation overlaps between ME/CFS, poliomyelitis and other non-polio enterovirus-related clinical outcomes as well as similarity in epidemic seasonality is further circumstantial evidence for a relationship between ME/CFS and enteroviruses. One possibility for the co-occurrence of polio and non-polio enteroviral outbreaks may be the environmental source of enteroviruses, which often are contaminated bodies of water. If sewage is contaminating water, consumers may be exposed to multiple types of enteroviruses.

To date, the body of research investigating enterovirus infections in relation to ME/CFS supports an increased prevalence of chronic or persistent infections in several ME/CFS patient cohorts. The majority of early EV-related investigations occurred within the UK from the 1970s to early 2000s, starting with serological tests but advancing to molecular methods including immunohistochemical detection of enterovirus viral capsid protein (VP1) and viral genome detection using RT-PCR (3, 13). Although a significant number of early papers provided evidence for an association of chronic enteroviral infections with ME/CFS, research into the enteroviral theory of disease etiology largely died out in the early 2000s with a few exceptions (7, 20). One reason that enteroviral research in ME/CFS has languished is the difficulty of detecting virus after time has passed following an acute infection. Furthermore, because enteroviral infections are frequent and common, a large fraction of the population will have serological evidence of exposures. Another issue is that reports of association of other pathogens and environmental stresses led to the concept that many different types of insults could result in ME/CFS. We are offering a critical evaluation of current literature that may lead to further inquiry into the role of EVs in ME/CFS.

In this review, we will first cover what is known about enteroviruses in relation to tissue tropism and ability to persist in a chronic infectious state. Emphasis will be put on the aspects of ME/CFS patient pathophysiology that are consistent with an active, chronic enterovirus infection. We will provide a critical review of studies that were attempting to identify chronic EV infections. The studies will be categorized based on the research methodology employed and special emphasis will be put on the sample types used and limitations to the chosen methods. We hope this review may help guide future viral-related studies by highlighting the tissue types and approaches most likely to provide insight into the hypothesis that enterovirus infections are initiating and/or perpetuating the disease state in ME/CFS.

## Background Regarding Enteroviruses

### Enterovirus Classification and Basic Molecular Biology

Although poliovirus is the most well-known enterovirus, it belongs to only one of 15 total enterovirus species including enterovirus species A-L and rhinovirus species A-C. Of the true enteroviruses, species A-D are known to have caused a wide spectrum of severe and deadly epidemics in humans (21, 22).

**Figure 1:**
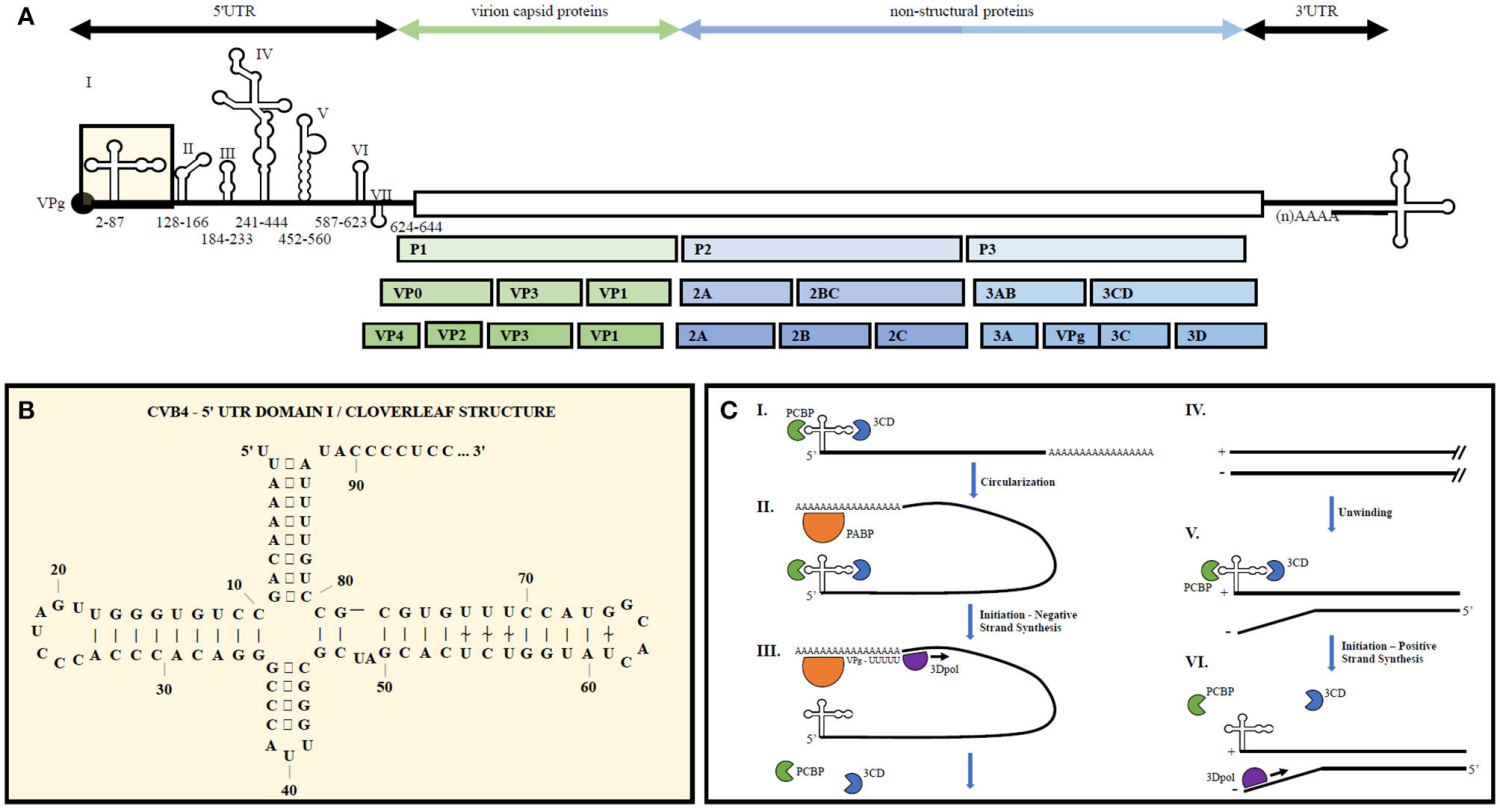
Representative enterovirus genome structure with emphasis on 5′UTR Domain I and genome replication. **(A)** Graphical depiction of EV genome as well as proteolytic processing to produce all structural and non-structural proteins. Number ranges indicate nucleotide positions for domains 1–7 in the 5′UTR of CVB4. **(B)** 2D illustration of CVB4 Domain I secondary structure. Numbers indicate nucleotide positions. **(C)** From (23) by license: Creative Commons Attribution 4.0 International. An integrated model for enterovirus replication. Negative-strand synthesis is initiated by circularization of the positive-strand genome *via* a protein-protein bridge through the interaction of the ternary complex at the 5′-end (3CD and PCBP bound to the cloverleaf structure) and PABP bound to the 3′-poly(A)tail (I. + II.). CRE-mediated VPg-pUpU acts as primer of the reaction and the polymerase 3D synthesizes the new negative-strand (III.), resulting in a double-stranded intermediate (RF) (IV.). The positive-negative duplex RNA intermediate unwinds, so that the cloverleaf structure at the 5′-end of the positive-strand can form. 3CD and PCBP bind to the cloverleaf to form a ternary complex, which, in turn, will initiate positive-strand synthesis on the 3′-end of the negative-strand (V.). The primer, VPg-pUpU, is recruited and binds to the 3′-terminal AA of the negative strand, and the new positive-strand is synthesized by the polymerase, 3D (VI.).

The enterovirus genome consists of a single stranded positive sense RNA molecule roughly 7.5 kb in length (see Figure 1). Upon translation *via* host cell machinery, one full length polypeptide is produced and then proteolytically cleaved into the polyprotein products PI, P2, and P3. P1 encodes four structural proteins, VP1-VP4, forming the non-enveloped virion capsid. P2 and P3 are proteolytically cleaved into 10 non-structural proteins including 2A to 2C, 3A to 3D as well as precursors 2BC, 3AB, and 3CD. Viral genomic RNA is capped on its 5′ end with the viral-encoded protein VPg (3B) instead of a methylated nucleotide cap structure.

Enteroviruses gain cellular entry through binding to host cell receptors and undergoing receptor-mediated endocytosis. Cellular receptors vary between EVs and include CD155/poliovirus receptor, integrins αvβ6 and αvβ3, ICAM-1, ICAM-5, CD55/decay accelerating factor, KREMEN1, coxsackievirus and adenovirus receptor (CAR), scavenger receptor B2, P-selectin glycoprotein ligand-1, sialylated glycan, heparan sulfate, neonatal Fc receptor, and annexin II (24–28).

Upon cellular entry, translation occurs following ribosome binding onto a type I internal ribosome entry site (IRES) located within the 5′UTR of the viral genome. Replication occurs *via* the viral encoded RNA-dependent RNA polymerase (3Dpol) which forms the negative sense RNA complement that is used to create additional positive sense RNA genomes (29). During active infection the ratio of positive to negative strands is roughly 100:1, whereas chronic infections display a ratio closer to 1:1 (7).

5′ and 3′ UTR secondary structures recruit both viral and host cell proteins to aid in viral translation and replication (30). The 5′UTR of EVs contains a cloverleaf secondary structure, termed Domain 1, as well as an internal ribosome entry site (IRES) containing six major stem-loop structures (see Figure 1). The 5′UTR is required for initiating both negative and positive strand RNA synthesis. The 3′UTR also contains important secondary structures, two predominant hairpin loops, that are the essential structure of the origin or replication for negative strand synthesis. Proteins bound to the 5′UTR interact with others bound to the genome's polyadenylation sequence at the 3′ end, thereby promoting viral genome circularization. Circularization allows the 3′UTR secondary structures to act as the initiation site for 3Dpol binding and at the origin of replication (31).

The viral encoded RNA polymerase is error-prone due to lack of a proof-reading mechanism, resulting in high mutation rates throughout enteroviral evolution. Furthermore, intra- and inter-typic genetic recombination may occur between enteroviruses, leading to increased genotypic plasticity. Enterovirus genomes frequently exhibit mosaic genomic sequences leading to a wide variety of genotypic and phenotypic diversity across enterovirus serotypes (32, 33).

### Enterovirus Carrier-State vs. Steady-State Persistent Infections

Persistent enteroviral infections are generally agreed to occur in two forms, termed carrier-state and steady-state persistence. In carrier-state infections, high levels of infectious virus are produced with infection limited to only a small proportion of cells. Alternatively, steady-state infections show all cells are simultaneously infected but viral replication is slowed, leading to a non-lytic phenotype with low viral copy numbers per cell. Both types of persistent viral infections are known to occur across human enteroviral species and have been linked to multiple clinical conditions (34–42).

Research on CVB4 infections of pancreatic ductal-like cells (PANC-1) and murine cardiac myocytes (HL-1) shows productive viral replication (10^6^-10^8^ PFU/ml) is restricted to a limited subpopulation of cells in culture and are therefore examples of carrier-state infections *in vitro*. PANC-1 cells exhibiting resistance to lysis *via* subsequent CVB4 superinfection were determined to be those PANC-1 cells with downregulated coxsackie adenovirus receptor (CAR) expression that became dominant in culture within several passages (43–45). These findings together illustrate the host cell's influence in the co-evolutionary balance between host and virus as the host attempts to limit viral infection from spreading *via* reduction in viral entry receptor expression (44). CVB1 infection of PANC-1 cells also demonstrates that CVB1 drives downregulation of cellular proteins involved in mitochondrial energy metabolism. Mitochondrial dysfunction, oxidative phosphorylation, fatty acid alpha- and beta-oxidation, citric acid cycle and leucine and valine degradation pathways were significantly enriched among downregulated proteins detected by mass spectrometry. Interestingly, further investigation into the mitochondrial networks of PANC-1 infected cells revealed differential changes in mitochondrial network morphology based on the CVB1 (ATCC vs. 10796) strain used to generate carrier-state infections. CVB1 strain 10796 produced fragmented mitochondrial networks whereas uninfected cells or those infected with CVB1 strain ATCC both showed filamentous mitochondrial networks. Proteomic analysis further supported these findings by revealing a significant downregulation in mitochondrial proteins involved in fusion processes including mitfusion-1, mitofusion-2, and mitochondrial dynamin-like GTPase OPA1 in the strain 10796-induced persistent infection model (46). In addition to support for carrier-state coxsackievirus-induced infections in the pancreas and heart, *in-vitro* infection of human astrocyte cells also suggests persistent coxsackievirus infection could occur in the central nervous system (CNS) (47).

Steady-state infections are characterized by all cells in culture having low levels of non-lytic viral replication. Low levels of viral replication lead to decreased viral-induced inhibition of host cell protein synthesis and thus lead to the non-lytic phenotype. To date, multiple studies have shown a subset of enterovirus serotypes, including coxsackieviruses and echoviruses, are able to produce low replicative steady-state infections without cytopathic effect. This phenomenon may be caused by a number of factors including but not limited to 5′UTR terminal deletions that lead to replication deficiencies or reduced type I interferon response elicitation, faulty virion capsid formation due to incomplete capsid polypeptide processing, and alternative EV RNA mutations that lead to abnormalities such as stable and atypical double-stranded RNA complex formation that inhibits further viral positive strand synthesis (48–51). In the context of ME/CFS, 5′UTR terminal deletions and/or atypical dsRNA complex formation are notable, as they have been shown to occur in a proportion of ME/CFS patient cohorts in multiple studies (52–54). In a number of cases, chronic diseases with some overlap in symptom constellation with ME/CFS show substantial evidence of disease involvement by persistent infection EV variants. These chronic diseases include idiopathic dilated cardiomyopathy (IDCM) (35), chronic inflammatory myopathy (36), insulin-dependent diabetes mellitus (37–39), post-polio syndrome (40, 41), and chronic CNS inflammation and lesions (42). For example, one study of EV positive-IDCM heart tissue detected a positive to negative strand ratio ranging from 2 to 20 (35), while another demonstrated EV-negative to positive strand ratios of 1–5 in infected heart tissue (55). Furthermore, the median viral load in heart tissue was assessed to be 287 EV RNA copies/μg of tissue. Such a low amount presents a significant challenge when trying to detect persistent enteroviral infections in difficult- to-sample/invasive secondary tissue screening sites. Low levels of viral replication result in EV RNA levels so small that they may be past the lower limit of detection (51).

In reviewing the outcomes of persistent *in-vitro* EV infections, it is clear that EVs with the ability to create carrier-state infections are able to produce cellular outcomes that may be relevant to ME/CFS pathophysiology in an EV variant-dependent manner. As mentioned above, specific CVB1 variants (CVB1 10796) disturb mitochondrial network morphology and lead to a downregulation of proteins relevant to mitochondrial energy metabolism. In regard to EVs that produce steady-state infections, Echovirus 6 and Enterovirus 72 (hepatitis A) are both shown to cause persistent steady-state infections *in-vitro* (48, 56, 57). Echovirus 6 is also shown to cause persistent *in-vivo* infections and is associated with neurological disorders of encephalitis and meningitis (58). Unfortunately, literature surrounding mitochondrial outcomes relating to these two viruses is bleak at best. Echovirus 6 infection of cultivated monkey kidney cells shows mitochondria retain their shape but information on mitochondrial enzymology and mitochondrial membrane potential is absent (59). Although there is a serious lack of literature pertaining to enterovirus steady-state infections and mitochondrial dysfunction, persistent Echovirus 6 infections are associated with non-lytic viral RNA and alterations in capsid protein production including unprocessed capsid polypeptide V0 (49). Considering the large number of interactions between enteroviral encoded and host proteins, it is reasonable to assume a downregulation and variation in viral encoded protein production during steady-state infections could lead to a mitochondrial dysfunction phenotype different and less extensive than seen in enterovirus carrier-state infections, acute infections, or cells without infection.

### Mitochondrial Abnormalities in ME/CFS Cells

There is recent literature that describes differences in immune cell metabolism between ME/CFS patients and controls (60–67). The relevance of these reports to possible dysfunction of mitochondria in tissues and organs is unclear. Immune cells alter their metabolism while responding to signals indicating a threat is present (68, 69). It is not known whether the altered mitochondrial metabolism is due to defective signaling or an appropriate immune response that is present in patients rather than healthy individuals, rather than an actual abnormality.

Early studies on 50 ME/CFS patient muscle biopsies found mitochondrial abnormalities described as branching and fusion of mitochondrial cristae upon ultrastructural examination in addition to swelling, vacuolation, myelin figures and secondary lysosomes indicating mitochondrial degeneration. The authors concluded their work was the first evidence that ME/CFS may be due to a mitochondrial disorder caused by a viral infection (70).

A few years later, right quadricep muscle biopsies from nine ME/CFS patients were assayed *via* electron microscopy, immunochemistry, mtDNA sequencing (as discussed earlier) and enzyme activity assays. The research group found mitochondrial structure abnormalities, inversion of the cytochrome oxidase/succinate dehydrogenase ratio and a reduction in some mitochondrial enzyme activities. The enzyme activity assay results indicate a reduction of the muscle oxidative property evaluated on multiple mitochondrial matrix enzymes including NADHtr, COX and succinate dehydrogenase. A reduction in mitochondrial enzyme activities was supported for cytochrome c oxidase and citrate synthetase as well (71).

Two recent studies found normal mitochondrial oxidative phosphorylation (oxphos) and normal respiratory chain complex activity compared to healthy controls. However, insight into mitochondrial oxidative phosphorylation was determined using plasma creatine kinase as a surrogate measure of oxphos in muscle (72, 73).

Another recent study used extracellular flux analysis *in vitro* to determine utilization of various substrates by skeletal muscle cells from patients vs. controls. This study found that muscle cells from ME/CFS patients had reduced oxphos in comparison to controls when supplied with glucose as a substrate, while no abnormalities were detected when cells were supplied with galactose or fatty acids (74).

Overall, the literature surrounding mitochondrial dysfunction in ME. CFS patients is suggestive of bioenergetic abnormalities that are within the realm of possible cellular outcomes based on the nature of the persistent viral infection. Varied findings pertaining to mitochondrial function in ME/CFS muscle biopsies may be due to sampling bias as latent enteroviral infections within secondary infection sites may not be uniform and therefore discovery of a cellular pathophysiology would only be found if the correct tissue location were interrogated.

### Enterovirus Cell and Tissue Tropism

Each enterovirus has a distinct cell and tissue level tropism that is governed by both host and viral factors, including cellular virus receptor availability, tissue-specific activity of IRES on viral RNAs, and innate immune antiviral activities such as interferon (IFN) response. Given these conditions, EVs as a whole display a wide spectrum of cell and tissue tropism leading to a wide array of disease outcomes. The diseases may appear as short-duration sicknesses such as the common cold and acute hemorrhagic conjunctivitis or may cause more serious diseases through infiltration into secondary infection sites such as organs, muscle or central nervous system (CNS), causing myocarditis, pericarditis, encephalitis, meningitis, pancreatitis, paralysis, and death (75).

CNS regulation of autonomic nervous system output occurs through multi-synaptic connections descending from the hypothalamus and midbrain to preganglionic neurons in the brainstem and spinal cord. The central autonomic system is further comprised of connections between a multitude of limbic system structures, such as the amygdala and hippocampus, to collectively regulate autonomic nervous system (ANS) outflow (76). The ANS is subdivided into the sympathetic, parasympathetic and enteric nervous systems, which act to control internal body processes such as blood pressure, heart and breathing rates, body temperature, digestion, metabolism, fluid retention, production of bodily fluids, urination, defecation, and sexual response (77).

ME/CFS patients have a number of pathophysiological traits that point to abnormalities in the ANS, including impaired blood pressure variability, orthostatic intolerance, high prevalence and severity of posturalorthostatic tachycardia syndrome (POTS), delayed gastric emptying, impaired thermoregulation in adolescent patients, loss of capacity to recover from acidosis on repeat exercise, abnormal cardiac output and altered brain characteristics in a wide variety of brain regions including the limbic system structures that govern the ANS (1, 78–81). These altered brain characteristics include reduced cerebral, brainstem, and cerebral cortex blood flow; impaired reciprocal connectivity between the vasomotor center, midbrain, and hypothalamus regions; increased neuroinflammation across widely distributed brain areas including but not limited to the hippocampus, thalamus, midbrain and pons; reduced cerebral glucose metabolism, and lower brain glutathione (1, 82–86). Many of the altered brain characteristics seen in ME/CFS patients are similarly reported in clinical cases associated with neurotropic enteroviruses. For instance, focal enterovirus encephalitis caused by coxsackievirus A3 is associated with focal hypoperfusion in the right frontal lobe that cleared upon patient recovery from the neurotropic enteroviral infection. This example case is largely similar to multiple SPECT studies indicating ME/CFS patients have significant hypo-perfusion in regions of the brain consistent with their patient-specific symptoms (87–92).

There is a diverse spectrum of tropisms for each enterovirus; some EVs are neurotropic in nature while others may be myotropic. Among human enterovirus families A-D, there exists a subset of EVs that are known to be neurotropic; these include EV71, multiple coxsackievirus group A members, all coxsackievirus group B members, poliovirus and EVD68, among many others. Not surprisingly, different neurotropic enteroviruses gain CNS access *via* alternative strategies and thus display distinct CNS tissue tropism. For example, poliovirus mainly infects and replicates in motor neurons in the anterior horn of the spinal cord, while EV71 primarily targets neuronal progenitor cells (NPSCs) and astrocytes (75). NPSC infection is particularly advantageous for viral dissemination, transmission, replication, and persistence. For instance, NPSC infection may expand CNS presence as the infected NPSCs differentiate into neuronal, astrocyte and oligodendrocyte lineages. Furthermore, NPSC migration following differentiation allows access into new CNS locations, and lastly, EV infection of NPSCs may trigger EV-specific genomic changes that allow the virus to persist in a latent state due to the quiescent cellular environment of non-activated NPSCs or NPSCs that have moved to a neuronal cell fate (93).

EVs gain access to the CNS through a diverse set of entry mechanisms including direct infection of brain microvascular endothelial cells, retrograde axonal transport following muscle infection, exosomal transport across the blood-brain barrier (BBB), and hitchhiking inside of migratory infected immune cells with BBB privilege (75). Infection outcomes can follow expected changes such as halting of host cell cap-dependent translational events and production of cytopathic effects causing tissue lesions. However, EVs may also establish a persistent/chronic infection producing atypical clinical outcomes, as may be the case in ME/CFS (75).

Several known EV-CNS infections display autonomic dysfunction symptoms reminiscent of those described in ME/CFS patients. Damage to the ANS is well-documented following poliovirus infection; postmortem histopathology routinely demonstrates damage to the reticular formation region of the brainstem whether or not the patient displayed spinal cord damage or paralysis (94). The reticular formation, a network of neurons located in the brainstem that project into the hypothalamus, thalamus, and cortex, plays a role as a cardiodepressor that lowers cardiovascular output. Post-polio syndrome (PPS) patients exhibit a high prevalence of hypertension and tachycardia while ME/CFS patients display high rates of POTS, which is accompanied by drop in blood pressure. The difference in autonomic dysfunction outcomes between ME/CFS and PPS patients may possibly be due to infection with genetically distinct EV serotypes with different neurotropism and thus different clinical manifestations. However, white matter brain lesions upon MRI, slowing of electroencephalography outputs, clinical impairment of attention, and abnormal hypothalamic pituitary adrenal axis function are shared between patients with PPS and those with ME/CFS (95). Nevertheless, there is a controversy about whether the reports of excess white matter lesions in ME/CFS patients are instead related to age, a misdiagnosis of neurological disorder, or due to major depression. A quantitative summary of rigorous data pertaining to white matter lesions in ME/CFS reported no significant increase in the lesions (96), but studies that use more advanced neuroimaging methods are needed.

Three ME/CFS post-mortem brain autopsy studies found enteroviral genomic RNA and VP1capsid protein in the hypothalamus, brainstem, cerebral cortex, medial temporal lobe, lateral frontal cortex, occipital lobe, and cerebellum (97–99). These findings provide additional support that a persistent EV infection within patient limbic and extra-limbic tissues is possible and could be driving the ANS dysfunction observed in ME/CFS patients.

CNS infections by other EVs such as EV71 and the group B coxsackieviruses result in ANS dysfunctions reminiscent of ME/CFS pathophysiology. EV71 brainstem encephalitis occasionally induces symptoms of ANS involvement including fluctuating blood pressure, tachycardia or bradycardia, hypertension or hypotension and respiratory distress. EV71 CNS-specific clinical manifestations include myoclonic jerk, polio-like syndrome, lethargy, limb weakness, altered mental status, encephalomyelitis, encephalitis, aseptic meningitis, and rhombencephalitis (100, 101). Of these EV71-related ANS/CNS clinical manifestations, altered blood pressure regulation, altered heart rate regulation, myoclonic jerk, lethargy, limb weakness and altered mental status are reported in ME/CFS patients, indicating a large overlap in symptom constellations between ME/CFS patients and neurotropic EV infections (102, 103).

To summarize, some serotypes of EVs exhibit CNS tropism and have the ability to produce persistent viral infections that result in atypical and distinct chronic clinical outcomes. Another complicating factor is the production of EV quasispecies, a population of EVs with subpopulations that consist of specific genotypic variants, each with genotypically-dependent functional characteristics. The proportion of the different quasispecies in the overall population dictates infection initiation, progression and dynamics of clinical presentation (93).

## Detection of Enteroviruses

### World Health Organization EV Surveillance Guidelines

The World Health Organization, in conjunction with the U.S. Centers for Disease Control, have published guidelines for enterovirus surveillance that details recommended procedures for specimen preservation as well as optimal methods for enterovirus detection and characterization. Although not all human enteroviruses can be propagated in cell culture, the guidelines state that multiple attempts should be made across a variety of cell lines including: primary African green, cynomolgus or rhesus monkey kidney cells (AGMK, CMK, RMK), rhesus monkey kidney (LLC-MK2), African green monkey kidney (Vero, BGMK, GMK), Madin Darby canine kidney (MDCK), human diploid cells lines (MRC-5, WI-38, SF), human embryonic kidney (HEK), human embryonic fibroblast (HEF), human epithelial carcinoma (HEp-2), and human rhabdomyosarcoma (RD) cells (104).

The guidelines further state the preferred and alternative sample types to use in cell culture inoculation depending on the clinical syndrome noted in patients. Based on the occurrence of encephalitis and respiratory clinical syndromes in a large proportion of ME/CFS cohorts, preferred sample types include brain tissue and broncho-alveolar lavage, with alternatively approved sample types, including cerebrospinal fluid (CSF), feces, throat swab, oropharyngeal swab, nasopharyngeal swab, and rectal swab (104).

### Approaches and Limitation of EV Detection Strategies Employed in ME/CFS Studies

Across the enterovirus and virus literature at large, a number of methodologies are used to detect the presence of enteroviral infection in patients. In the early years of virus detection, biological approaches such as serological testing and cell culture methods were employed. Isolation *via* cell culture requires patient samples to be inoculated into enterovirus-susceptible cell lines and then examined periodically for the presence of viral-induced changes such as cytopathic effect (CPE), which is described as cells becoming rounded, refractile and shrinking before detaching from the cell surface. The identity of the isolated virus was then confirmed/typed *via* tests such as neutralization of infectivity with serotype-specific antisera or immunochemistry using fluorescent antibodies. The main disadvantages to cell culture are that inoculation depends on quality of the patient sample and requires variable and sometimes extended time periods to allow detection (105, 106). Some enteroviruses, especially persistent enterovirus variants, do not produce CPE in cell culture. Without CPE, screening for viral nucleic acid or protein would be necessary.

Serological testing is confounded by several factors. First, enteroviruses often produce clinical disease before the appearance of antibodies, making their detection retrospective. Furthermore, enteroviruses and rhinoviruses have extensive antigenic heterogeneity and lack cross-reacting antigens, so that many different antigens would be needed to detect anti-EV antibodies (105–107). Virus antigen detection can be achieved both by immunohistochemical detection and ELISA. Viral antigens such as VP1 exhibit sequence similarity between serotypes, which is an advantage in detection of enteroviruses, but also means that serotype identification is not feasible solely from reaction with a VP1 antigen. Commercial labs with serological tests for EVs are far from comprehensive. For instance, the Enterovirus IgG/IgA/IgM ELISA kits sold *via* Virotech Diagnostics detects 14 (CVA9, CVA16, CVB2, CVB4, CVB5 and Echo 5, 11, 15, 17, 22, 23, 25, 33) of the roughly 120 known EV serotypes (108). The Enterovirus Antibody Panel lab test provided by ARUP Laboratories similarly detects 14 EV serotypes (CVA9, CVB1-6, Echo 6, 7, 9, 11 and 30, poliovirus types 1 and 3) although the serotypes differ slightly (109). Negative detection of EVs *via* these commercially available serological tests does not conclusively eliminate the possibility of an EV infection. Other companies, such as SERION Diagnostics and Immuno-Biological Laboratories also sell enterovirus-specific ELISA kits but with the added benefit of using recombinant antigens. The recombinant antigens are made from conserved and subtype specific domains across a subset of human enteroviruses and are therefore likely to demonstrate antigens for all known human enteroviruses. These kits have an increased comprehensive nature, but a positive detection cannot reveal exactly which EV serotype is the culprit in question.

A serological method for detection of antibodies to enteroviruses that has not yet been employed in ME/CFS is the peptide array, which is comprised of tiled peptides corresponding to a virus family. Such an array designed to probe human herpesviruses has been used to compare ME/CFS patients to healthy controls and individuals with other diseases (110). An enterovirus peptide array was successfully used to detect antibodies against EV-68 in some samples of cerebrospinal fluid and serum from patients with acute flaccid myelitis (111).

The most popular detection method for identification of enteroviruses is RT-PCR, with amplification directed at conserved regions of the enterovirus genome, including those encoding the 5′UTR, 3Dpol and VP1. VP1 is the region of choice to conduct enterovirus typing. However, low sequence similarity amidst the approximately 120 enterovirus serotypes means that no one primer set is robustly comprehensive so that RT-PCR methods would have a lower chance of identifying novel EV serotypes than unbiased sequencing. RT-PCR experiments that use primers directed at the 5′UTR of enteroviruses can be problematic if the enterovirus contains mutations within the primer binding region, as is known to happen during persistent infection. Traditional RT-PCR approaches have reduced ability to identify novel enteroviruses that could be etiological agents in new diseases.

Northern blots using sequences complementary to EV genomic regions to detect viral RNA in a gel are similarly confounded by a lack of comprehensiveness, as the probe sequence might fail to hybridize to EV serotypes that have sufficient variation in targeted sequences. For greater sensitivity and breadth, many researchers have instead used an unbiased RNAseq approach to detect enterovirus nucleic acids in patient samples. In terms of disadvantages, RNAseq is expensive and requires significant read depth in sequencing to identify low copy transcripts among the sea of nucleic acids that are being sequenced. Capture approaches have been developed to enhance sensitivity and increase breadth of viral detection (112–114) (Table 1).

**Table 1:** Compilation of enterovirus-specific ME/CFS studies listed by tissue type and sub-grouped based on EV detection methodology.

	**Positive studies**	**Total studies**	**Prevalence in positive cohorts**
Blood	20	24	8–100%
Serological test	16	20	8–90%
PCR	4	5	18–100%
RNAseq	0	2	N/A
Muscle	8	11	13–53%
PCR	6	9	13–100%
Northern blot	4	4	21–50%
VP1 immunohistochemistry	0	1	N/A
Throat swab	1	1	17%
PCR	1	1	17%
Stomach tissue	2	2	82%
PCR	1	1	37%
VP1 immunohistochemistry	2	2	82%
dsRNA immunohistochemistry	1	1	64%
Heart Tissue	1	1	N/A
PCR	1	1	N/A
Cerebrospinal fluid	1	2	N/A
Tissue culture	1	1	50%
EV IgG ELISA	0	1	N/A
Brain tissue	3	3	N/A
PCR	2	2	N/A
VP1 immunohistochemistry	2	2	N/A
Feces	2	4	22–25%
PCR	0	1	N/A
Tissue culture	2	4	22–25%
Electron microscopy	0	1	N/A

*Many studies utilize multiple detection methods resulting in the total number of studies not equaling the number of studies based on each method*.

### Critical Review of EV Detection in ME/CFS by Method Used

#### Tissue Culture Reports

To date, ME/CFS studies reporting the use of tissue culture for EV detection have used CSF and feces in 1 and 4 studies, respectively (115–118). The singular CSF study reported two EV infections in a cohort of 4 patients, while the 4 fecal studies reported an increased EV infection prevalence in 2 of 4 studies, with cohorts ranging from a 22–25% prevalence across patient cohorts (Table 1).

Although the prevalence of EV infections in these studies was generally shown to be significantly increased compared to healthy control cohorts, limitations in patient sample types and cell culture models may have led to findings that underrepresent the prevalence of EV infections in patient cohorts. Of the five cell culture studies, one study used only one cell type (115), 3 studies used two cell types (115–117) and one study used three cell types (118).

The most comprehensive study, which utilized three cell culture types, included green monkey kidney cells, RD cells and HeLa cells, which together supply a diversity of enterovirus receptors including CAR, CD155 and DAF. These cultures therefore detect a wide diversity of enteroviruses although the system is still not totally comprehensive. No enterovirus-positive fecal samples were found within a cohort of 12 ME/CFS patients (118) when the triple-cell culture method was used. EVs may be absent in these patients, but lack of detection might also be attributed to the presence of an enterovirus that uses an alternative receptor as well as the low likelihood of detecting EV infections in the stool samples of chronically ill patients with persistent infections in secondary sites such as muscle and brain tissue. Furthermore, the investigators were searching for CPE, and EVs present in chronic infections commonly undergo genetic changes which reduce CPE. An example of the inadequacy of CPE is a report that inoculated cell cultures were negative for CPE production in human fetal lung fibroblast and tertiary monkey kidney cell cultures but were nevertheless positive upon RT-PCR (119).

Two studies utilized Hep-2, VERO, and monkey kidney tissue cultures for identification of enterovirus from CSF and feces from 4 and 76 patients, respectively. Innes (115) identified enterovirus in 2 of 4 CSF samples and one of 4 feces samples (115). Yousef et al. (116) found that 17/76 patients tested positive for enterovirus infection while only 2/30 controls tested positive (116).

Studies reporting the absence of enterovirus infections in ME/CFS patient cohorts using tissue culture approaches had small sample sizes and incomprehensive cell culture systems. Small sample sizes along with the fact that EVs harboring 5′UTR deletions do not produce CPE means that no definitive conclusion can be made about the absence of EVs from the data in these studies. Furthermore, fecal samples usually identify only acute enterovirus infections and not chronic ones that might be in secondary infection sites. Nevertheless, some studies that screened suboptimal sample types with culture methods did find an increased prevalence of EV infections, which might have been due to inclusion of patients who were still in the acute phase of illness.

#### Serological Testing for EVs

A wide variety of serological tests for detection of EVs have been developed. Studies between the 1970s and late 1990s that screened for EV infections in ME/CFS patients largely focused on serological testing. The diversity of testing employed in a total of 20 serological-based ME/CFS studies included neutralization, complement fixation, micro-metabolic inhibition, ELISA, indirect immunofluorescence, and VP1 antigen detection tests. In total, 16 of the 20 studies found an increased prevalence of CVB signals in ME/CFS cohorts with positive findings ranging from 8 to 90% compared to the positive findings in healthy control cohorts that ranged from 0 to 65% (Table 1) (115–118, 120–135).

The vast majority of studies evaluated the presence of antibodies directed only against CVB enteroviruses, with a few exceptions in which echo30- and echo9-directed IgG antibodies were screened *via* ELISA (118). A notable study was performed in 1997, in which neutralization tests for 11 enteroviruses (CVB1-6 and echo 6, 7, 9, 11, 30) found that 100 out of 200 tested patients had elevated enteroviral titers (136).

Although serological testing in ME/CFS cohorts generally shows an increase in the prevalence of EV antibodies, the findings often lack clinical specificity as a high prevalence of EV antibodies are found in the general population from previous exposure. In a retrospective study, it cannot be known whether the enterovirus infection occurred before or after ME/CFS disease onset without having paired sera from both time periods.

#### Immunohistochemistry to Detect EV Capsid Proteins and dsRNA

The enteroviral capsid protein VP1 is commonly used for identification of enteroviral virions in ME/CFS patient tissues. In total, 5 studies have used this technique on a variety of patient sample types, including muscle, gastrointestinal, and brain tissue (Table 1, Supplementary Table 1) (20, 54, 98, 99, 137). Of these, 4 out of 5 studies identified the presence of VP1 capsid proteins in patient tissue. The muscle tissue study did not detect VP1 staining in samples of a cohort of 30 ME/CFS patients, despite RT-PCR signals that indicated the presence of EV-RNA in 13 of the same 30 patients. The authors suggested that the difference in PCR and VPI immunochemistry resulted from persistent but latent enteroviral infection in patient muscle tissues, in which no detectable amount of virion particles were being produced (137).

The remaining 4 studies showed positive VP1 staining in both gastrointestinal and brain tissues (20, 54, 98, 99). Gastrointestinal samples exhibited positive staining rate of 82% in two patient cohorts (*n* = 165, *n* = 416). Comparative cohorts for these two studies were healthy controls (*n* = 34) and patients with functional dyspepsia (FD) (*n* = 66), which displayed a positive VP1 staining rate of 20 and 83%, respectively (20, 99). Both the ME/CFS and FD patient cohorts showed dsRNA staining for 64 and 63% of patients, respectively (54). Because persistent/chronic EV infections with reduced CPE and viral replication typically have a 1:1 ratio between enteroviral positive and negative RNA strands, finding a high rate of dsRNA in patient tissues indicates the likely presence of persistent enteroviral infections. One study found VP1 in fibroblasts of small blood vessels in the cerebral cortex and in a small fraction of glial cells in brain (98), while another detected VP1 instead in the pontomedullary junction, medial temporal lobe, lateral frontal cortex, occipital lobe, cerebellum and midbrain (99).

#### Molecular Approaches to Detect EV Infections

We identified 24 reports of the use of either Northern Blot (*n* = 4) (52, 138–140), RT-PCR (*n* = 18) (20, 53, 97, 99, 117, 118, 132, 134, 137, 141–145) or RNAseq (*n* = 2) (146, 147) across multiple sample types including blood, feces, muscle, brain, heart, gastrointestinal tissue and throat swabs. In a few cases, a single publication used RT-PCR on multiple sample types; thus, there are 20 independent studies amongst the 24 reports. Seventeen of the twenty publications report detection of EVs in patient samples or indicate an increased prevalence of EV infections compared to control cohorts (Table 1, Supplementary Table 1).

The 4 Northern blot studies used muscle tissue biopsies and were all positive for viral RNA, indicating an EV prevalence between 21 and 50% in ME/CFS with control cohorts showing a prevalence between 0 and 1% (52, 138–140). The two RNAseq studies were negative for the presence of EV in blood, whether or not blood was taken before or after an exercise stress that exacerbated subject symptoms (146, 147). While RNAseq is a more comprehensive approach to enterovirus detection than Northern blots, these studies cannot be directly compared since one used muscle tissue and the other assayed blood samples.

With regard to EV studies that applied RT-PCR methods, 5 of the 17 reports indicated no significant difference in EV prevalence between ME/CFS and control cohorts. The 5 reports were performed on peripheral blood leukocytes (132), muscle tissue (118, 141, 142), and feces (119). A list of all 8 PCR approaches/methods, indicating the primer sets employed in RT-PCR experiments, was first compiled, and then each PCR set was examined for its effectiveness for detection of all 117 known EV serotypes (Table 2, Supplementary Tables 2, 3). *In-silico* PCR was run with conservative allowances (1 mismatch and no mismatches within 2 base pairs of the 3′end) as well as less conservative allowances (4 mismatches with mismatches being allowed on the 3′end) to give a range of possible experimental results, given that one *in-silico* PCR experiment does not likely represent the true *in-vitro* PCR outcomes. The less conservative *in-silico* experiments resulted in predicted binding to multiple locations, sometimes over 15 locations along an EV genome, and thus were not likely to represent results that would be gained from an actual experiment. Examining the conservative *in-silico* PCR experiments that used 1 mismatch and 0 allowed mismatches within the 3′end of the primer (Supplementary Table 2 indicated methods 1, 3, 5, 7, and 8 are low in their comprehensive nature with 52, 50, 21/0, 39, and 65 EVs being amplified out of 117, respectively. Interestingly, four (118, 119, 132, 141) of the five (118, 119, 132, 141, 142) studies indicating a lack of EV presence by RT-PCR used primer sets from methods 1, 7, and 8, which amplify 44, 33, and 56% of known human enteroviruses, respectively. Therefore, an EV infection could have been present and simply escaped detection due to the primer sets employed. One study reported 20.8% (*n* = 48) of the ME/CFS cohort to have detectable EVs compared to 0% (*n* = 29) of controls even though method 5 was used, in which round 2 PCR primers amplify 0% of EVs under conservative PCR parameters and 3% of EVs under less conservative parameters. Either that reported primer sequence does not function as expected in the *in-silico* PCR or the particular EV that was detected in these patients is one of the few able to be observed with method 5 primers. Poor *in-silico* PCR amplification using method 5 was caused by the primer OL253 (5′-GATACTYTGAGCNCCCAT-3′) used in the second round PCR. First round primers, OL252 and OL68, as well as second round primer OL24 had binding rates to the EV serotypes with only OL253 lacking *in-silico* hybridization. Overall, RT-PCR experiments with low rates of positive *in-silico* PCR amplification are strongly correlated with publications indicating insignificant differences in EV prevalence between controls and patients (Table 2, Supplementary Tables 2, 3).

**Table 2:** *In-silico* PCR amplification results using primers reported throughout enterovirus-specific ME/CFS publications.

**References**	**PCR primers and probes**	**No. amplified EVs: 1 mismatch**	**No. amplified EVs: 4 mismatches**
(132, 141)	1: EP1, EP4 and EP2 (probe)	52/117	92/117
(142)	2: EP1, EP4	85/117	112/117
(53, 134, 144, 145)	3: 1 (EP1, EP4), 2(P6, P9)	(85/117), (50/117)	(112/117), (112/117)
(97)	4: Primer 2, Primer 3 and Probe	102/117	112/117
(143)	5: 1(OL252, OL68), 2(OL24, OL253)	(21/117), (0/117)	(111/117), (3/117)
(20, 99, 137)	6: RNC2, NC1, E2 and Probe	89/117	108/117
(117)	7: 1(Primer 1, Primer 4), 2(Primer 2, Primer 3 and Probe)	(77/117), (39/117)	(103/117), (75/117)
(118)	8: 1(Primer 1, Primer 2), 2(Primer 3, Primer 4)	(65/117), (65/117)	(110/117), (110/117)

*Nested RT-PCR studies (methods 3, 5, 7, and 8) are reported as PCR amplification results for both rounds of PCR (1st round), (2nd round). Values indicate number of EVs with a positive PCR amplification out of 117 total EVs tested for amplification. In-silico PCR was performed using Geneious Prime v2019.1.1. Primer design uses a modified version of Primer3 v2.3.7. Primers could bind anywhere on sequence (1 mismatch): in-silico PCR testing allowed 1 mismatch to be tolerated between the primer and its complementary site on the EV transcript. The 1 allowed mismatch was not allowed to occur within 2 base pairs of 3′ end. The 1 mismatch PCR parameter therefore indicates an in-silico PCR amplification with low forgiveness for incomplete complementarity and reduces the likelihood of off target amplification events (4 mismatches): in-silico PCR testing allowed 4 mismatches to be tolerated between the primer and its complementarity site on the EV transcript. All 4 mismatches could occur at any site within the primer and EV transcript complementarity region. The 4 mismatch PCR parameter therefore indicates an in-silico PCR amplification with high forgiveness for incomplete complementarity and increases the likelihood of both on and off target amplification events*.

**Figure 2:**
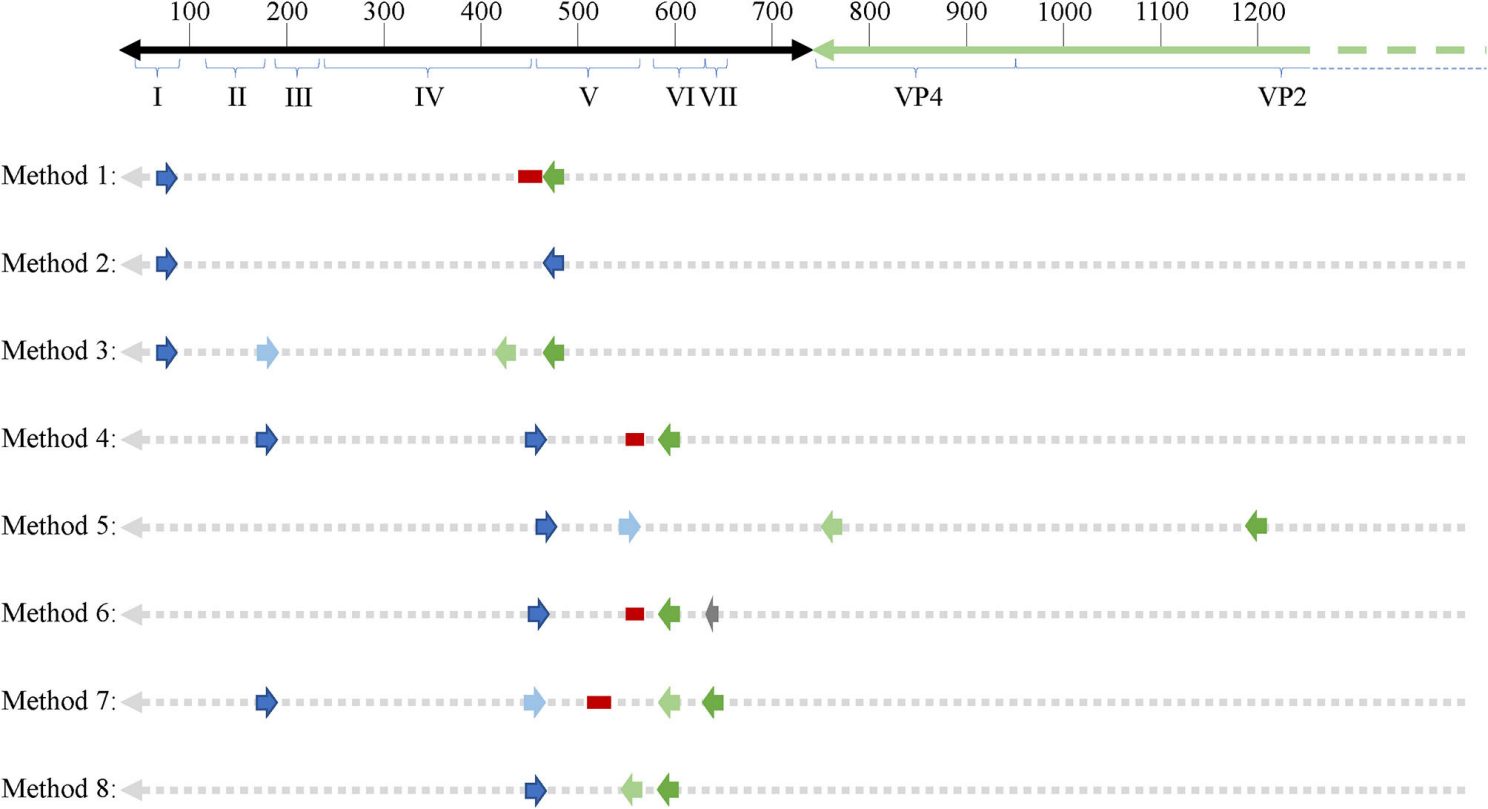
Schematic showing primer binding sites across an enteroviral genome. 5′UTR domains are indicated by roman numerals. Numbers on top of the representative genome indicate nucleotide position. Forward and reverse primers as well as probes (if applicable) are indicated across all 8 PCR methodologies used across enterovirus-related ME/CFS studies. Dark blue arrows indicate forward primers, dark green arrows indicated reverse primers, second round primers used in nested PCR approaches are indicated by light blue (forward) and light green (reverse) arrows. Red bars indicate probes and the one gray arrow indicates a primer used in the primary reverse transcription step.

As mentioned earlier, EVs are known to exhibit mutations in the 5′UTR that result in replication deficiencies. Interestingly, all 8 PCR methodologies used primer pairs targeting the 5′UTR with the exception of method 5 whose reverse primers target the VP4 and VP2 genomic regions (see Figure 2). This is an important consideration as patients infected with EV variants exhibiting 5′UTR deletions may not be successfully targeted by the primer sets employed across these PCR methodologies. In conclusion, PCR studies aimed at identifying EVs in ME/CFS have been crippled by the use of incomprehensive primer sets that target potentially deleted portions of the viral genome.

## Discussion

Multiple aspects of the ME/CFS pathophysiology, especially related to autonomic dysfunction, are reminiscent of chronic neurotropic enterovirus-related diseases and clinical outcomes. This fact, in conjunction with the enterovirus-like seasonality of ME/CFS epidemics, often occurring in spatiotemporal incidence with known poliomyelitis epidemics of the time, gives strong justification for the conclusion that enteroviruses have been etiological agents in ME/CFS outbreaks.

Many ME/CFS patients in a variety of studies indicate a viral-like illness immediately preceded their ME/CFS symptoms. However, surveys also indicate that patients ascribe their onset to a variety of other reasons, including emotional stress, life events, recent travel, accidents, toxic substances, or mold (148, 149). However, some of these events and exposures could merely be coincidental and actually be due to an enteroviral infection that was unnoticed or very mild, given that many enteroviral infections are asymptomatic (150). The COVID19 pandemic has made it obvious that persistent symptoms can arise from mild or asymptomatic infections (151). Were the existence of SARS CoV-2 not known, many of the individuals with long-lasting symptoms of COVID 19 might readily have ascribed their mysterious illness to some other factor than viral infection.

Post-acute viral syndromes may not all fit the diagnostic criteria recommended by the U.S. Institute of Medicine (IOM) for ME/CFS (152), as the victims of a number of viral infections have not been thoroughly investigated over long time periods. Further, even the definition of ME/CFS or SEID itself may be lumping together disparate phenomena (153). The last report on the 2003 SARS outbreak patients exhibiting long-term symptoms followed them up to only 2 years later (154). At this writing, individuals who contracted SARS-CoV-2 and did not recover completely have been ill no more than 14 months, and many are displaying not only symptoms required for the IOM definition of ME/CFS, but additional ones, suggesting that further study may differentiate them at the molecular/biochemical level from individuals with pre-2020 ME/CFS. New information from ongoing studies of the consequences of COVID19 may indicate that the definition of ME/CFS will need to be refined to distinguish it from post-acute SARS-CoV-2 syndrome, even though a number of symptoms overlap. Notably, Gulf War Illness victims have symptoms that overlap with ME/CFS, but a number of studies are able to distinguish them from individuals with ME/CFS who did not participate in Gulf War era military activities (155–158).

A relatively small number of viruses have been identified as possible triggers for ME/CFS, making the concept held by some, that “any virus” can lead to ME/CFS, unsupported by evidence. One of the few studies of viral triggers of fatiguing syndromes is being carried out in Australia, namely the Dubbo study of post-infective fatigue syndromes, which follows individuals with diagnosed Ross River virus, Epstein-Barr Virus (EBV), as well as Q fever (a bacterial rather than viral infection) (9, 159–161). Given the geographic limitation to Ross River virus exposure, it is not likely that it is a major cause of ME/CFS worldwide.

There appears to be a special relationship between herpesvirus infection and ME/CFS, as recently reviewed (8, 162). Whether this is actually a relationship between enteroviral and herpesviral infection is not known. Several studies have documented that a certain percentage of people who contract mononucleosis from Epstein-Barr virus infection will still be ill 6 months or more, exhibiting symptoms diagnostic of ME/CFS (163, 164). Surveys often indicate that a proportion of patients believe their ME/CFS followed an acute case of mononucleosis or other type of herpesvirus infection (149, 164, 165). However, given that enteroviruses are known often to cause mild or asymptomatic infections, it is possible that individuals who report ME/CFS after mononucleosis or other herpesviral infections may have also had an inciting enterovirus infection before or after the herpesvirus infection. In fact, one may speculate that an undetected enteroviral infection could make an individual more susceptible to symptomatic cases of EBV infection, for example. Most individuals are infected with EBV as children, yet a number of patients have reported an adult-onset EBV infection as triggering their ME/CFS. Perhaps these adult cases are actually mis-diagnosed reactivated infections. Indeed, there are several reports of reactivated herpesvirus infections in ME/CFS patients (166, 167). Furthermore, a few studies have discovered impaired immunological response to EBV in ME/CFS patients (168, 169). Is this impaired response due to a prior or ongoing enteroviral infection? Whether or not herpesviruses may incite ME/CFS or merely take advantage of immune disruptions caused by enteroviral infections, they may contribute to the symptoms of the illness, and may prevent recovery, as illustrated by a subset that improves upon anti-herpesvirus drug treatment (170–172).

Our review emphasizes that EV-related ME/CFS literature indicates that some patients exhibit chronic enteroviral infection. Furthermore, our review highlights a number of experimental weaknesses (cohort size, tissue type interrogated, methodological approach, etc.) that exist across the EV-ME/CFS literature for studies both supporting or opposing increased EV infection prevalence in ME/CFS patients vs. healthy controls. Those studies that do not support an increased prevalence of EV infections in ME/CFS patient cohorts using RT-PCR are especially confounded with issues related to incomprehensive RT-PCR primer design. Considering that the majority of patient samples interrogated have been collected from patients in the chronic stage of illness, too few studies have been directed at more appropriate secondary infection tissue sites that would give insight into the possibility of persistent myotropic or neurotropic enteroviruses. Indeed, the majority of studies interrogating muscle tissue and all studies we have identified interrogating brain tissue or cerebrospinal fluid *via* PCR or tissue culture have found detectable signs of EV infection. It is evident that more research must be conducted in order to determine whether or not the majority of pre-2020 ME/CFS cases have arisen from EV infection. At the time of this writing, there have been a number of anecdotal reports of individuals experiencing remission of long-term COVID19 symptoms after receiving anti-SARS COV2 vaccines. Such a therapy will not be possible for any ME/CFS patients whose illness is due to chronic infection unless the persistent virus is identified.

Moving forward, studies aimed at identifying chronic EV infections in ME/CFS patients need to consider quality and types of samples to interrogate as well as methodological approaches to employ. The key samples suggested to interrogate further would include brain tissue, cerebrospinal fluid, and muscle biopsy samples. As of now, we could identify only 5 studies reporting on the assessment of either brain (*n* = 3) or cerebrospinal fluid (*n* = 2) and these studies are either on individual patients or cohorts of up to 7. Muscle biopsies have been chosen as the source of patient tissue sample in a total of 11 identified studies, but problems in RT-PCR primer design, small cohorts and few biological tissue replicates means the conclusions of the 8 studies reporting an increased EV prevalence in ME/CFS cohorts may be underrepresenting the true prevalence. Furthermore, the 3 studies indicating a lack of increased prevalence may have been unable to identify the EV serotype in question.

In terms of methodological approaches, RT-PCR with optimal primer sets and or RNAseq with target capture enrichment should be utilized as the methodology of choice for EV detection specifically. Both experimental approaches may be modified to allow detection of both positive and negative strand viral transcripts and are also advantageous in their ability to detect low copy number transcripts. Targeted RNAseq has the increased benefit of being completely comprehensive for the enteroviral family, allowing complete genomic sequencing as well as an increased likelihood of identifying novel EV serotypes possibly at play in an illness such as ME/CFS whose inciting pathogen remains unidentified.

## Author Contributions

AO'N: examined efficacy of published primers *in silico*. AO'N and MH: reviewed literature and wrote the paper. Both authors contributed to the article and approved the submitted version.

## Conflict of Interest

The authors declare that the research was conducted in the absence of any commercial or financial relationships that could be construed as a potential conflict of interest.
