# Supplementary material for: The Enterovirus Theory of Disease Etiology in Myalgic Encephalomyelitis/Chronic Fatigue Syndrome: A Critical Review
Source: Front Med (Lausanne). 2021 Jun 18;8:688486. doi: 10.3389/fmed.2021.688486 (PMC8253308; doi:10.3389/fmed.2021.688486)
Supplement: Supplementary file 1 [file Table_1.docx]

Supplementary Material

Supplementary Table 1. Complete list of Enterovirus-related ME/CFS studies consulted in preparation of this review. Publications are grouped by tissue type interrogated and methodological approach is given. (% ME/CFS +) indicates percentage of the given ME/CFS patients who were enterovirus positive. (% Controls +) indicates percentage of the given controls who were enterovirus positive. (EV ME/CFS > EV Controls) indicates if the ME/CFS cohort was found to have a statistically significant higher prevalence of enterovirus infections compared to controls.

|  | | **Publication** | | **Method** | | **% ME/CFS +** | **% Controls +** | | | **EV ME/CFS > EV Control** |
| --- | --- | --- | --- | --- | --- | --- | --- | --- | --- | --- |
| Blood | | S.G.B. Innes (1970) | | Serological Testing - CVB Neutralization Test | | 50% (n=4) | N/A | | | + |
|  |  | B.D. Keighley, E.J. Bell (1983) | | Serological Testing - CVB Neutralization Test | | 80% (n=20) | N/A | | | + |
|  |  | K.G. Fegan et al. (1983) | | Serological Testing - CVB Neutralization Test | | 82% (n=22) | N/A | | | + |
|  |  | B.D. Calder, P.J. Warnock (1984) | | Serological Testing - CVB Neutralization Test | | 47% (n=81) | N/A | | | + |
|  |  | B.D. Calder et al. (1984) | | Serological Testing - CVB Neutralization Test | | 46% (n=140) | 25% (n=100) | | | + |
|  |  | E.J. Bell, R.A. McCartney (1984) | | Serological Testing - CVB Neutralization Test | | 41% (n=52) | 4% (n=950) | | | + |
|  |  | I.E. Salit (1985) | | Serological Testing - CVB Neutralization Test | | 8% (n=50) | N/A | | | + |
|  |  | Behan et al. (1987) | | Serological Testing - CVB Neutralization Test | | 70% (n=50) | N/A | | | + |
|  |  |  | | Serological Testing - CVB IgM ELISA | | 12% (n=50) | N/A | | | + |
|  |  | G.E. Yousef et al. (1988) | | Serological Testing - VP1 Antigen Test | | 51% (n=87) | 0% (n=30) | | | + |
|  |  |  | | Serological Testing - CVB IgM ELISA | | 74% (n=87) | 0% (n=30) | | | + |
|  |  | E.J. Bell et al. (1988) | | Serological Testing - CVB Neutralization Test | | 12.5% (n=247) | 4% (n=950) | | | + |
|  |  |  | | Serological Testing - CVB IgM ELISA | | 37% (n=290) | 9% (n=500) | | | + |
|  |  | D. Halpin, S. Wessely (1989) | | Serological Testing - VP1 Antigen Test | | 30% (n=30) | 12% (n=43) | | | + |
|  |  | P.M.J. Wilson et al. (1989) | | Serological Testing - CVB Neutralization Test | | 44% (n=39) | N/A | | | + |
|  |  |  | | Serological Testing - CVB IgM ELISA | | 46% (n=39) | N/A | | | + |
|  |  | E.G. Dowsett et al. (1990) | | Serological Testing - CVB Neutralization Test | | 50% (n=205) | N/A | | | + |
|  |  |  | | Serological Testing - CVB IgM ELISA | | 31% (n=124) | N/A | | | + |
|  |  | N.A. Miller et al. (1991) | | Serological Testing - CVB IgM ELISA | | 24.4% (n=217) | 22.6% (n=217) | | | **-** |
|  |  |  | | Serological Testing - IgG micrometabolic inhibition method | | 56.2% (n=217) | 55.3% (n=217) | | | **-** |
|  |  | J.W. Gow et al. (1991) | | Serological Testing - CVB Neutralization Test | | 20% (n=60) | 15% (n=41) | | | + |
|  |  | A.L. Landay et al. (1991) | | Serological Testing - Indirect Immunofluorescence CVB4 | | 90% (n=63) | 65% (n=40) | | | + |
|  |  | J.W. Gow et al. (1991) | | PCR - periphreal blood leukocytes | | 16% (n=20) | 16% (n=20) | | | **-** |
|  |  | C.M. Swanink et al. (1994) | | Serological Testing - VP1 Antigen Test | | 67% (n=24) | 77% (n=22) | | | **-** |
|  |  |  | | Serological Testing - Complement Fixation Test (geometric mean (median) titer) | | 51.3 (32) | 53.6 (32) | | | **-** |
|  |  |  | | Serological Testing - CVB IgG ELISA | | 21% (n=24) | 23% (n=22) | | | **-** |
|  |  |  | | Serological Testing - CVB IgM ELISA | | 21% (n=24) | 18% (n=22) | | | **-** |
|  |  |  | | Serological Testing - CVB IgA ELISA | | 8% (n=24) | 14% (n=22) | | | **-** |
|  |  | G.B. Clements et al. (1995) | | nested PCR | | 41% (n=88) | 2% (n=126) | | | + |
|  |  | C. Nairn et al. (1995) | | Serological Testing – CVB Neutralization Test | | 34% (n=100) | 41% (n=100) | | | - |
|  |  |  | | nested PCR | | 42% (n=100) | 9% (n=100) | | | + |
|  |  | D.N. Galbraith et al. (1995) | | nested PCR | | 18% (n=238) | 2% (n=130) | | | + |
|  |  | D. Buchwald (1996) | | Serological Testing – CVB Neutralization Test | | 20% (n=508) | N/A | | | + |
|  |  | G. Lindh et al. (1997) | | Serological Testing - echo30, CVB5 and echo9 IgG ELISA | | 0% (n=7) | N/A | | | **-** |
|  |  | D.N. Galbraith et al. (1997) | | nested PCR + sequencing of PCR product | | 100% (n=8) | N/A | | | + |
|  |  | Jerome Bouquet (2017) | | RNAseq | | 0% (n=25) | 0% (n=25) | | | **-** |
|  |  | Jerome Bouquet (2019) | | RNAseq after CPET | | 0% (n=14) | 9% (n=11) | | | **-** |
| Muscle Tissue | | L.C. Archard et al. (1988) | | Northern Blot | | 21% (n=96) | 0% (n=4) | | | + |
|  |  | L. Cunningham et al. (1990) | | Northern Blot | | 50% (n=8) | 0% (n=152) | | | + |
|  |  | J.W. Gow et al. (1991) | | PCR | | 53% (n=60) | 15% (n=41) | | | + |
|  |  | L. Cunningham et al. (1991) | | Northern Blot | | 24% (n=140) | 0% (n=152) | | | + |
|  |  | N.E. Bowles et al. (1993) | | Northern Blot | | 26% (n=158) | 1% (n=152) | | | + |
|  |  | J.W. Gow et al. (1994) | | PCR | | 26.4% (n=121) | 19.8% in OND (n=101) | | | **-** |
|  |  | F. McGarry et al. (1994) | | PCR | | 100% (n=1) | N/A | | | + |
|  |  | A. McArdlle et al. (1996) | | PCR | | 0% (n=34) | 0% (n=10) | | | **-** |
|  |  | G. Lindh et al. (1997) | | semi-nested PCR | | 0% (n=29) | N/A | | | **-** |
|  |  | R.J. Lane et al. (2003) | | nested PCR | | 20.8% (n=48) | 0% (n=29) | | | + |
|  |  | F. Douche-Aourik et al. (2003) | | PCR | | 13% (n=30) | 0% (n=29) | | | + |
|  |  |  | | VP1 immunohistochemistry | | 0% (n=30) | 0% (n=29) | | | **-** |
| Throat Swabs | D.N. Galbraith et al. (1995) | | nested PCR | | 17% (n=175) | | | N/A | + | |
|  |  |  |  |  |  |  |  |  |  |  |
|  |  |  |  |  |  |  |  |  |  |  |
|  |  |  |  |  |  |  |  |  |  |  |
| Stomach Tissue | J.K. Chia, A.Y. Chia (2008) | | RT-PCR ELISA - based on Rotbart's Method | | 37% (n=24) | | | <1% (n=21) | + | |
|  |  | | VP1 immunohistochemistry | | 82% (n=165) | | | 20% (n=34) | + | |
|  | J.K. Chia et al. (2015) | | mAb against dsRNA | | ME/CFS w/ FD 64% (n=416) | | | FD alone 63% (n=66) | + | |
|  |  | | VP1 immunohistochemistry | | ME/CFS w/ FD 82% (n=416) | | | FD alone 83% (n=66) | + | |
| Heart Tissue | F. McGarry et al. (1994) | | PCR | | 100% (n=1) | | | N/A | + | |
| CSF | S.G.B. Innes (1970) | | H.Ep.II tissue culture, monkey kidney tissue culture | | 50% (n=4) | | | N/A | + | |
|  | G. Lindh et al. (1997) | | Serological Testing - echo30, CVB5 and echo9 IgG ELISA | | 0% (n=7) | | | N/A | **-** | |
| Brain Tissue | F. McGarry et al. (1994) | | PCR | | 100% (n=1) | | | N/A | + | |
|  | J. Richardson (2011) | | VP1 immunohistochemistry | | 100% (n=1) | | | N/A | + | |
|  | J.K. Chia et al. (2015) | | VP1 western blot | | 100% (n=1) | | | N/A | + | |
|  |  | | RT-PCR | | 100% (n=1) | | | N/A | + | |
| Feces | S.G.B. Innes (1970) | | monkey kidney tissue culture | | 25% (n=4) | | | N/A | + | |
|  | G.E. Yousef et al. (1988) | | VERO and Hep-2 tissue culture | | 22% (n=76) | | | 7% (n=30) | + | |
|  | C.M. Swanink et al. (1994) | | nested PCR | | 4% (n=24) | | | 0% (n=22) | **-** | |
|  |  | | human fetal lung fibroblast and tertiary monkey kidney cell tissue culture | | 0% (n=24) | | | 0% (n=22) | **-** | |
|  | G. Lindh et al. (1996) | | green monkey kidney cells, RD cells and HeLa cells tissue culture | | 0% (n=12) | | | N/A | **-** | |
|  |  | | electron microscopy | | 0% (n=12) | | | N/A | **-** | |

Supplementary Table 2. Complete in-silico PCR results. Mismatches = 1. 0 allowed mismatches within 2 base pairs of the 3’end. ** indicates faulty primer reported in publication.

| **Primer/Probe** |  |  |  |
| --- | --- | --- | --- |
| Method 1 (Detect 44% of Human Enteroviruses) |  | EP1, EP4 and EP2 |  |
| EPI: 5'-CGGTACCTTTGTGCGCCTGT-3' | EVA (25) | 8/25 |  |
| Probe EP2: 5'-TATTGAGCTAGTTGGTAGTCCTCCGG-3' | EVB (63) | 44/63 |  |
| EP4: 5'-TTAGGATTAGCCGCATTCAG-3' | EVC (24) | 0/24 |  |
|  | EVD (5) | 0/5 |  |
|  | total | 52/117 |  |
|  |  |  |  |
| Method 2 (Detect 73% of Human Enteroviruses) |  | EP1 and EP4 |  |
| EPI: 5'-CGGTACCTTTGTGCGCCTGT-3' | EVA (25) | 16/25 |  |
| EP4: 5'-TTAGGATTAGCCGCATTCAG-3' | EVB (63) | 59/63 |  |
|  | EVC (24) | 9/24 |  |
|  | EVD (5) | 1/5 |  |
|  | total | 85/117 |  |
|  |  |  |  |
| Method 3 (Detect 73% of Human Enteroviruses) |  | EP1 and EP4 | P9 and P6 |
| EPI: 5'-CGGTACCTTTGTGCGCCTGT-3' | EVA (25) | 16/25 | 13/16 |
| EP4: 5'-TTAGGATTAGCCGCATTCAG-3' | EVB (63) | 59/63 | 37/59 |
|  | EVC (24) | 9/24 | 0/9 |
|  | EVD (5) | 1/5 | 0/1 |
|  | total | 85/117 | 50/117 |
|  |  |  |  |
| Method 4 (Detect 68%, 87% of Human Enteroviruses) |  | Primer 1, Primer 3 and Probe | Primer 2, Primer 3 and Probe |
| Primer 1: 5'-CAAGCACTTCTGTTTCCCCGG-3' | EVA (25) | 21/25 | 24/25 |
| Primer 2: 5'-TCCTCCGGCCCCTGAATGCG-3' | EVB (63) | 44/63 | 58/63 |
| Primer 3: 5'-ATTGTCACCATAAGCAGCCA-3' | EVC (24) | 15/24 | 17/24 |
| Probe 5'-AAACACGGACACCCAAAGTA-3' | EVD (5) | 0/5 | 3/5 |
|  | total | 80/117 | 102/117 |
|  |  |  |  |
|  |  |  |  |
|  |  |  |  |
| **Primer/Probe** |  |  |  |
| Method 5 (Detect 18%, 0% of Human Enteroviruses) |  | OL252 and OL68 | OL24 and OL253 |
| OL252: 5'-GGCCCCTGAATGCGGCTAA-3' | EVA (25) | 0/25 | 0/0 |
| OL68: 5'-GGGACCTTCCACCACCANCC-3' | EVB (63) | 20/63 | 0/20 |
| OL24: 5'-CTACTTTGGGTGTCCG-3' | EVC (24) | 0/24 | 0/0 |
| OL253: 5'-GATACTYTGAGCNCCCAT-3' ** | EVD (5) | 1/5 | 0/0 |
|  | total | 21/117 | 0/117 |
|  |  |  |  |
| Method 6 (Detect 76% of Human Enteroviruses) |  | RNC2, NC1, E2 and Probe |  |
| RT primer: RNC2 - 5'-CACCGGATGGCC-3' | EVA (25) | 19/25 |  |
| NC1: 5'-CTCCGGCCCCTGAATGCG-3' | EVB (63) | 53/63 |  |
| E2: 5'-ATTGTCACCATAAGCAGCCA-3' | EVC (24) | 16/24 |  |
| probe S08: 5'-AAACACGGACACCCAAAGTA-3' | EVD (5) | 1/5 |  |
|  | total | 89/117 |  |
|  |  |  |  |
| Method 7 (Detect 62%, 33% of Human Enteroviruses) |  | Primer 1 and Primer 4 | Primer 2, Primer 3 and Probe |
| Primer 1: 5'-CAAGCACTTCTGTTTCCCCGG-3' | EVA (25) | 18/25 | 12/18 |
| Primer 4: 5'-CACCGGATGGCCAATCCA-3' | EVB (63) | 40/63 | 26/40 |
| Primer 2: 5-TCCTCCGGCCCCTGAATGCG-3' | EVC (24) | 14/24 | 1/14 |
| Primer 3: 5'-ATTGTCACCATAAGCAGCCA-3' | EVD (5) | 0/5 | 0/0 |
| Probe: 5'-TGTGTCGTAACGGGCAACTCTGCAGCGGAA-3' | total | 72/117 | 39/117 |
|  |  |  |  |
| Method 8 (Detect 56%, 56% of Human Enteroviruses) |  | Primer 1 and Primer 2 | Primer 3 and Primer 4 |
| Primer 1: 5’-AGTCCTCCGGCCCCTGAATGCGGCTA-3’ | EVA (25) | 10/25 | 10/10 |
| Primer 2: 5’-ACTGGCTGCTTATGGTGACA-3’ | EVB (63) | 48/63 | 48/48 |
| Primer 3: 5’-AGTCCTCCGGCCCCTGAATGCGGCTA-3’ | EVC (24) | 6/24 | 6/6 |
| Primer 4: 5’-ACTACTTTGGGTGTCCGTGTT-3’ | EVD (5) | 1/5 | 1/1 |
|  | total | 65/117 | 65/117 |

Supplementary Table 3. Complete in-silico PCR results. Mismatches = 4. Mismatches allowed within 3’end. ** indicates faulty primer reported in publication. Supplementary Table 2. Complete in-silico PCR results. Mismatches = 4. Mismatches allowed within 3’end. ** indicates faulty primer reported in publications.

| **Primer/Probe** |  |  |  |
| --- | --- | --- | --- |
| Method 1 (Detect 79% of Human Enteroviruses) |  | EP1, EP4 and EP2 |  |
| EPI: 5'-CGGTACCTTTGTGCGCCTGT-3' | EVA (25) | 18/25 |  |
| Probe EP2: 5'-TATTGAGCTAGTTGGTAGTCCTCCGG-3' | EVB (63) | 62/63 |  |
| EP4: 5'-TTAGGATTAGCCGCATTCAG-3' | EVC (24) | 11/24 |  |
|  | EVD (5) | 1/5 |  |
|  | total | 92/117 |  |
|  |  |  |  |
| Method 2 (Detect 96% of Human Enteroviruses) |  | EP1 and EP4 |  |
| EPI: 5'-CGGTACCTTTGTGCGCCTGT-3' | EVA (25) | 23/25 |  |
| EP4: 5'-TTAGGATTAGCCGCATTCAG-3' | EVB (63) | 63/63 |  |
|  | EVC (24) | 23/24 |  |
|  | EVD (5) | 3/5 |  |
|  | total | 112/117 |  |
|  |  |  |  |
| Method 3 (Detect 96%, 96% of Human Enteroviruses) |  | EP1 and EP4 | P9 and P6 |
| EPI: 5'-CGGTACCTTTGTGCGCCTGT-3' | EVA (25) | 23/25 | 23/23 |
| EP4: 5'-TTAGGATTAGCCGCATTCAG-3' | EVB (63) | 63/63 | 63/63 |
|  | EVC (24) | 23/24 | 23/23 |
|  | EVD (5) | 3/5 | 3/3 |
|  | total | 112/117 | 112/117 |
|  |  |  |  |
| Method 4 (Detect 93%, 96% of Human Enteroviruses) |  | Primer 1, Primer 3 and Probe | Primer 2, Primer 3 and Probe |
| Primer 1: 5'-CAAGCACTTCTGTTTCCCCGG-3' | EVA (25) | 23/25 | 24/25 |
| Primer 2: 5'-TCCTCCGGCCCCTGAATGCG-3' | EVB (63) | 61/63 | 61/63 |
| Primer 3: 5'-ATTGTCACCATAAGCAGCCA-3' | EVC (24) | 22/24 | 23/24 |
| Probe 5'-AAACACGGACACCCAAAGTA-3' | EVD (5) | 3/5 | 4/5 |
|  | total | 109/117 | 112/117 |
|  |  |  |  |
|  |  |  |  |
|  |  |  |  |
| **Primer/Probe** |  |  |  |
| Method 5 (Detect 95%, 3% of Human Enteroviruses) |  | OL252 and OL68 | OL24 and OL253 |
| OL252: 5'-GGCCCCTGAATGCGGCTAA-3' | EVA (25) | 22/25 | 0/22 |
| OL68: 5'-GGGACCTTCCACCACCANCC-3' | EVB (63) | 63/63 | 0/63 |
| OL24: 5'-CTACTTTGGGTGTCCG-3' | EVC (24) | 23/24 | 2/23 |
| OL253: 5'-GATACTYTGAGCNCCCAT-3' ** | EVD (5) | 3/5 | 1/3 |
|  | total | 111/117 | 3/117 |
|  |  |  |  |
| Method 6 (Detect 92% of Human Enteroviruses) |  | RNC2, NC1, E2 and Probe |  |
| RT primer: RNC2 - 5'-CACCGGATGGCC-3' | EVA (25) | 24/25 |  |
| NC1: 5'-CTCCGGCCCCTGAATGCG-3' | EVB (63) | 61/63 |  |
| E2: 5'-ATTGTCACCATAAGCAGCCA-3' | EVC (24) | 21/24 |  |
| probe S08: 5'-AAACACGGACACCCAAAGTA-3' | EVD (5) | 2/5 |  |
|  | total | 108/117 |  |
|  |  |  |  |
| Method 7 (Detect 88%, 64% of Human Enteroviruses) |  | Primer 1 and Primer 4 | Primer 2, Primer 3 and Probe |
| Primer 1: 5'-CAAGCACTTCTGTTTCCCCGG-3' | EVA (25) | 22/25 | 15/22 |
| Primer 4: 5'-CACCGGATGGCCAATCCA-3' | EVB (63) | 60/63 | 59/60 |
| Primer 2: 5-TCCTCCGGCCCCTGAATGCG-3' | EVC (24) | 18/24 | 1/18 |
| Primer 3: 5'-ATTGTCACCATAAGCAGCCA-3' | EVD (5) | 3/5 | 0/3 |
| Probe: 5'-TGTGTCGTAACGGGCAACTCTGCAGCGGAA-3' | total | 103/117 | 75/117 |
|  |  |  |  |
| Method 8 (Detect 94%, 94% of Human Enteroviruses) |  | Primer 1 and Primer 2 | Primer 3 and Primer 4 |
| Primer 1: 5’-AGTCCTCCGGCCCCTGAATGCGGCTA-3’ | EVA (25) | 24/25 | 24/24 |
| Primer 2: 5’-ACTGGCTGCTTATGGTGACA-3’ | EVB (63) | 61/63 | 61/61 |
| Primer 3: 5’-AGTCCTCCGGCCCCTGAATGCGGCTA-3’ | EVC (24) | 21/24 | 21/21 |
| Primer 4: 5’-ACTACTTTGGGTGTCCGTGTT-3’ | EVD (5) | 4/5 | 4/4 |
|  | total | 110/117 | 110/117 |
|  |  |  |  |
|  |  |  |  |
|  |  |  |  |
|  |  |  |  |
